# The Relationship Between Availability and Changes to Perceived Workplace Support and Their Impact on the Mental Health, Well-being and Burn-Out of Healthcare Professionals (HCP): Insight and Mitigating Strategies From the CoPE-HCP Cohort Study

**DOI:** 10.1192/bjo.2022.215

**Published:** 2022-06-20

**Authors:** Imrana Siddiqui, Jaya Gupta, Iris McIntosh, Christina Komodromos, Thomas Godec, George Collett, Sher Ng, Carmela Maniero, Sotiris Antoniou, Rehan Khan, Vikas Kapil, Mohammed Y. Khanji, Ajay K. Gupta

**Affiliations:** 1 Wellbeing Hub, Newham Training Hub, London, United Kingdom; 2 NHS North East London CCG, London, United Kingdom; 3 Woodgrange Medical Practice, London, United Kingdom; 4 Barnet, Enfield and Haringey Mental Health NHS Trust, London, United Kingdom; 5 Camden & Islington Foundation Trust, London, United Kingdom.; 6 William Harvey Research Institute, Queen Mary University of London, London, United Kingdom; 7 Barts Heart Centre, St. Bartholomew's Hospital, Barts Health NHS Trust, London, United Kingdom; 8 The Royal London Hospital, Barts Health NHS Trust, London, United Kingdom; 9 Newham University Hospital, Barts Health NHS Trust, London, United Kingdom; 10 National Heart and Lung Institute, Imperial College London, London, United Kingdom

## Abstract

**Aims:**

To examine the relationship between self-reported level of workplace support (WS) and various mental health outcomes in HCPs and non-HCPs at different time-points during the COVID-19 pandemic, and to examine whether improved WS is associated with improved mental health outcomes over time. Lastly, to identify what support healthcare professionals (HCPs) perceive to be most helpful.

**Methods:**

Cohort survey study at baseline (July-September 2020) and follow-up (approximately four months later).

**Setting**

HCPs working in primary or secondary care, from UK and other countries, and non-HCP controls from primarily London-based universities.

**Participants**

1574 HCPs and 147 non-HCPs (academic and research staff at London-based universities). The inclusion criteria for the study were: 1) aged 18 or older, 2) electronic consent given, and 3) identified as HCP or non-healthcare academic staff or self-declared non-HCPs.

**Main outcome measures**

Presence of generalized anxiety disorder (assessed using the GAD-7), clinical insomnia (ISI), major depressive disorder (PHQ-9), well-being (SWEMWBS), and burnout (emotional exhaustion and depersonalization; EEDP2Q). Qualitative data exploring what support HCPs perceive as most useful was gathered using free-text inputs.

**Results:**

At baseline and follow-up, consistently, compared to those who felt unsupported, those who felt supported had significantly reduced risk (odds) of generalized anxiety disorder (baseline: 59% [95% CI of OR, 0.29 to 0.57], follow-up: 41% [0.38 to 0.92]), clinical insomnia (51% [0.34 to 0.69], 66% [0.20 to 0.55]), major depressive disorder (58% [0.31 to 0.58], 54% [0.31 to 0.74]), emotional exhaustion (65% [0.26 to 0.46], 61% [0.27 to 0.56]) and depersonalization (58% [0.28 to 0.61], 68% [0.21 to 0.50]).

At follow-up, self-reported improved WS (vs. baseline) was associated with significantly improved GAD-7 (adjusted difference. −1.73 [-2.54 to −0.91]), ISI (-0.96 [-1.88 to −0.04]), PHQ−9 (-1.32 [-2.16 to −0.49]), SWEMWBS (0.97 [0.37 to 1.57]) and EEDP2Q (burnout) (-1.30 [-1.82 to −0.79]) scores, independent of baseline level of support.

Five themes were identified constituting WS: ‘managerial support’ was the largest sub-theme.

**Conclusion:**

A consistent association was observed between level of WS and the mental health of HCPs and non-HCPs. Improved WS was associated with improved mental health scores over a four-month period during the pandemic.

